# Galectin-3: a novel biomarker of glycogen storage disease type III

**DOI:** 10.1038/s41420-025-02452-6

**Published:** 2025-04-14

**Authors:** Lucille Rossiaud, Quentin Miagoux, Manon Benabides, Océane Reiss, Louisa Jauze, Margot Jarrige, Hélène Polvèche, Edoardo Malfatti, Pascal Laforêt, Giuseppe Ronzitti, Xavier Nissan, Lucile Hoch

**Affiliations:** 1 Université Paris-Saclay, Université d’Evry, Inserm, IStem, UMR861, Corbeil-Essonnes, France; 2 IStem, CECS, Corbeil-Essonnes, France; 3 IStem, CECS, The Research and Innovation Team, Corbeil-Essonnes, France; 4 Genethon, Evry, France; 5 Université Paris-Saclay, Univ Evry, Inserm, Genethon, Integrare research Unit UMR_S951, Evry, France; 6 Reference Center for Neuromuscular Disorders, APHP Henri Mondor University Hospital, Créteil, France; 7 Université Paris Est Créteil, Inserm, U955, IMRB, Créteil, France; 8 Neurology Department, Nord/Est/Île-de-France Neuromuscular Reference Center, FHU PHENIX, AP-HP, Raymond-Poincaré Hospital, Garches, France

**Keywords:** Predictive markers, Metabolic disorders, Transcriptomics, Induced pluripotent stem cells

## Abstract

Glycogen storage disease type III (GSDIII) is a rare genetic disorder leading to abnormal glycogen storage in the liver and skeletal muscle. In this study, we conducted a comparative gene expression analysis of several in vitro and in vivo models and identified galectin-3 as a potential biomarker of the disease. Interestingly, we also observed a significant decrease in galectin-3 expression in mice treated with an AAV gene therapy. Finally, galectin-3 expression was studied in muscle biopsies of GSDIII patients, confirming its increase in patient tissue. Beyond the identification of this novel biomarker, our study offers a new perspective for future therapeutic developments.

## Introduction

Glycogen storage diseases are genetic metabolic disorders involving dysfunctions in glycogen synthesis, glycogen degradation, or the glycolytic pathway [1]. Among them, glycogen storage disease type III (GSDIII) is an autosomal recessive glycogen debranching disorder due to mutations in the amylo-α-1, 6-glucosidase, 4-α-glucanotransferase gene (*AGL*) [1]. Although over-represented in some specific subpopulations such as Inuit Canadians, Faroese, Tunisians or North African Jewish in Israel, the incidence is estimated at 1 in 100,000 in the United States [2–5]. Molecularly, the *AGL* gene encodes the glycogen debranching enzyme (GDE) and its deficiency results in abnormal glycogen (limit dextrin) accumulation, mainly in the liver, heart, and skeletal muscles. Human muscle biopsies show a typical and consistent vacuolar myopathy, characterized by multiple and large vacuoles filled with PAS-positive material corresponding to normally branched glycogen and ultrastructural autophagic figures [6]. Clinically, the first manifestations of the disease appear in childhood with growth retardation and acute liver symptoms with severe fasting hypoglycemia, hepatomegaly, liver enzymes increase [7] and liver fibrosis [8]. Liver symptoms gradually subside from adolescence onwards, but liver complications such as liver cirrhosis, hepatocellular adenomas, or carcinomas may occur in a small proportion of patients (11% in the Sentner cohort [7]) and may lead to liver transplantation [7, 9]. During childhood, muscle symptoms progressively appear and worsen with age. Skeletal muscle impairments include myopathy with muscle weakness and exercise intolerance and can evolve towards loss of ambulation [7, 9, 10]. Heart involvement consists of hypertrophic cardiomyopathy which can become symptomatic with rare cases of heart failure [7, 9–11]. Other rare features such as reduced bone mineral density [7, 12, 13], polycystic ovary syndrome [7, 14] and type 2 diabetes [7] are also reported. Among these symptoms, skeletal muscle involvement has been identified in GSDIII adult patients as the major burden of the disease and the most important target for potential therapy [10, 15].

To date, no curative treatment is available and only dietary management is recommended to patients. During childhood, the clinical focus is the control of hypoglycemia with frequent and small meals of complex carbohydrates [16–18]. Later, dietary management shifts to the prevention of long-term complications with low-carbohydrates, high-protein, and sometimes high-fat diet, which brings improvements in the heart but with less efficiency in skeletal muscles [19–23]. Among the different therapeutic strategies under development, the most promising one is gene replacement therapy to re-express GDE. Nevertheless, the major limitation of this approach is the large size of the human GDE cDNA (5 kb), which cannot fit into a single AAV. To overcome this issue, a dual overlapping AAV expressing the entire human GDE cDNA was previously reported demonstrating a preclinical efficacy. More recently, a bacterial debranching enzyme (pullulanase), shorter enough to be encapsidated in a single AAV, showed muscle or liver correction depending on the vector and promoter used in the mouse model [24]. Although efficient in the short term, this led to a loss of correction over time due to the immune response against the bacterial enzyme [25]. Very recently, a single AAV containing a human mini-GDE transgene was shown to rescue heart and muscle impairments in a GSDIII mouse model and to reduce glycogen content in an in vitro human muscular model developed by our team [26]. Another study demonstrated that the combination of gene therapy with rapamycin treatment in mouse synergizes to restore both cytosolic and lysosomal glycogen degradation pathways in the muscle [27]. Although these studies demonstrated that AAV-based gene therapy was efficient to rescue glycogen accumulation and to reduce muscular dysfunctions, authors also highlighted the need to identify new predictive biomarkers to monitor the disease progression in the muscle.

Several engineered mouse models [24, 28–31] and a natural canine model [32] that faithfully recapitulate the features of GSDIII patients have been reported, but until recently there was no readily available and tissue-specific in vitro model in a human context. To overcome this issue, we recently reported that skeletal myotubes (skMt) derived from patient or CRISPR/CAS9-induced pluripotent stem cells recapitulated the glycogen accumulation phenotype under glucose starvation conditions [33, 34]. More recently, our group reported that this model could be used as a platform to optimize and evaluate new gene therapy approaches [26, 33] but also to decipher the molecular changes induced by the combination of two treatments, i.e., rapamycin and a dual-AAV gene therapy [27]. Here, we used these different in vitro and in vivo models to identify a novel biomarker for GSDIII impairments in muscles. We report the up-regulation of galectin-3 (GAL3) in three GSDIII models i.e., human skMt, mouse muscles, and patient muscle biopsies. We also report that GAL3 dysregulation was normalized following gene therapy confirming its potential use as a marker of GSDIII disease progression in muscle and to inform on the efficacy of future therapies.

## Results

### Phenotypical characterization of CTRLs and GSDIII^Patients^ skMt

**Fig. 1:**
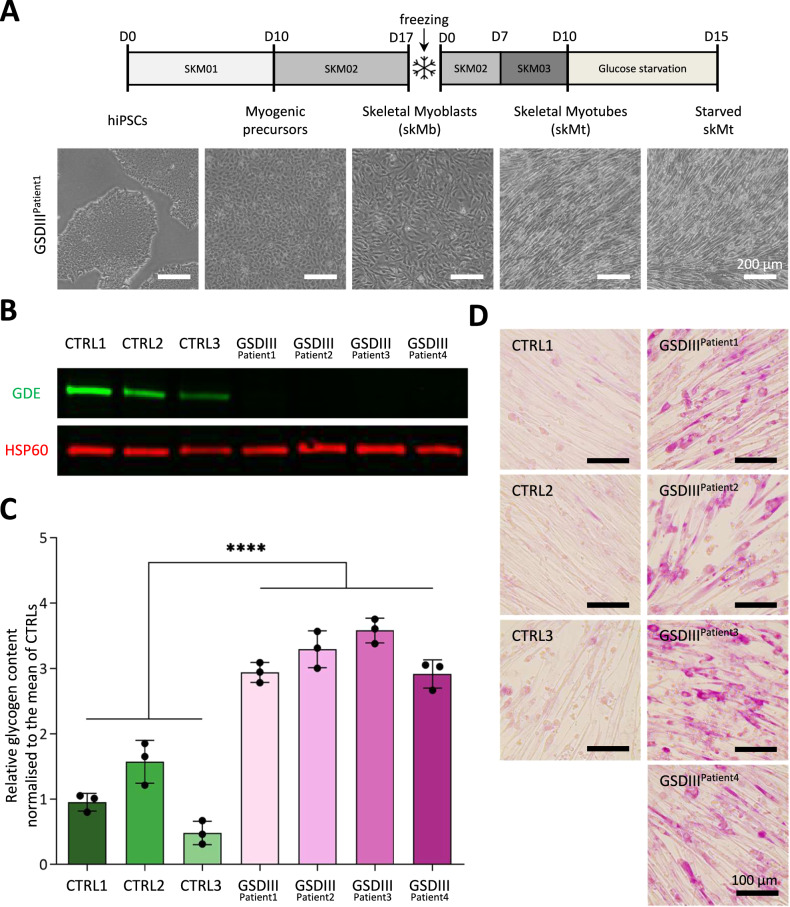
Phenotypical characterization of CTRLs and GSDIII^Patients^ skMt. All the experiments were performed after 5 days of glucose starvation. **A** Schematic representation of the skeletal myogenic differentiation protocol and phase contrast microscopic images of cell morphology along differentiation. hiPSCs were differentiated into myogenic precursors in 10 days of incubation with SKM01 medium and then into skMb in 7 days of incubation with SKM02 medium until frozen. SkMb were thawed and incubated in SKM02 medium for 7 days to reach confluence and transferred to SKM03 medium for 3 additional days to obtain skMt. For glucose starvation experiments, skMt was incubated in a glucose-free medium for 5 additional days. Scale bar = 200 μm. **B** Western Blot analysis of GDE (green) and HSP60 (red) was used as a loading control. **C** Glycogen content revealed by enzymatic test. Statistical analysis was performed using an unpaired *t*-test comparing the pool of GSDIII^Patients^ vs the pool of CTRLs cell lines. ****p value < 0.0001; *n* = 3 per cell line. Data are shown as mean ± standard deviation. **D** Glycogen content revealed by periodic acid Schiff staining. Scale bar = 100 μm.

To model GSDIII, 3 CTRLs, and 4 GSDIII^Patients^ hiPSC lines were differentiated into skMt following a 3-step protocol [35]. Briefly, hiPSCs initiate their differentiation into myogenic precursors in the SKM01 medium, then mature into skeletal muscle myoblasts (skMb) in the SKM02 medium and terminally differentiate into skMt in the SKM03 medium (Fig. 1A). A full characterization of the differentiation process of the CTRL1, CTRL2, CTRL3, and GSDIII^Patient1^ hiPSCs was performed in previous studies [33, 36]. To follow the differentiation process of GSDIII^Patient2^, GSDIII^Patient3^, and GSDIII^Patient4^ hiPSCs, quantitative PCR was performed showing a decrease of pluripotency markers (*OCT4* and *NANOG*) and an increase of myogenic markers (*DESMIN, MYOD, MYOG*, and *DP427M*) throughout differentiation (Supplementary Fig. S1A). Immunostaining confirmed the presence of myogenic markers (TITIN, DESMIN, and MYOG) at a protein level in GSDIII^Patient2^, GSDIII^Patient3^, and GSDIII^Patient4^ skMt (Supplementary Fig. S1B). To investigate the phenotype of CTRLs and GSDIII^Patients^ skMt, a western blot analysis was performed showing the absence of GDE protein in GSDIII^Patients^ skMt compared to CTRLs skMt (Fig. 1B). In order to reveal a differential capacity to consume glycogen content, our previous study demonstrated the need to starve skMt with a culture medium containing no glucose [33]. After 5 days of glucose starvation (Fig. 1A), significantly higher glycogen content in GSDIII^Patients^ skMt compared to CTRLs skMt was measured using an indirect enzymatic assay (Fig. 1C) and confirmed by PAS staining (Fig. 1D).

### Bulk RNA sequencing analysis of GSDIII^Patients^ vs CTRLs skMt

**Fig. 2:**
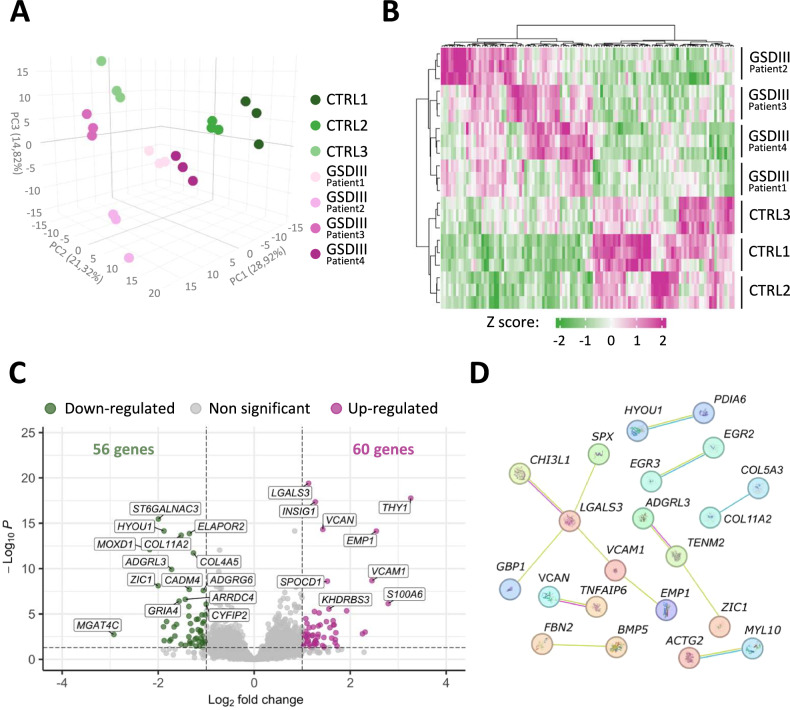
Bulk RNAseq analysis of GSDIII^Patients^ vs CTRLs-starved skMt. The bulk RNAseq was performed after 2.5 days of glucose starvation. **A** PCA represents the variability of all conditions and their replicates. **B** Heatmap representation of Hierarchical Clustering Analysis (HCA) generated on the DEGs. **C** Volcano plot representation of down (green) and up (pink) DEGs. Corrected *p* value < 0.05. |fold change| > 2. **D** Gene interaction network of the DEGs generated with String-db software. Only genes described in the literature with direct protein interaction and a medium confidence >0.4 are represented.

To study the skeletal muscle pathophysiological mechanisms of GSDIII, we performed a bulk RNA sequencing of the three CTRLs and the four GSDIII^Patients^ generated skMt. All skMt were deprived of glucose for 2.5 days in order to sequence skMt when glycogen catabolism was active and glycogen differentially accumulated between CTRLs and GSDIII^Patients^ skMt. As a control of this experimental setup, periodic acid Schiff staining was performed revealing a differential glycogen consumption in skMt derived from the representative cell lines CTRL1 and GSDIII^Patient1^ (Supplementary Fig. S2). Principal component analysis (PCA) of the entire dataset, and a heatmap of differentially expressed genes (DEGs), revealed a separation between CTRLs and GSDIII^Patients^ skMt in terms of mRNA expression profile (Fig. 2A, B). Despite this separation, PCA also showed heterogeneity between the different patient lines. Among the 116 DEGs, 56 were up-regulated and 60 were down-regulated in GSDIII^Patients^ skMt compared to CTRLs skMt (Fig. 2C). Validation of the deregulation of the 4 most down-regulated and the 4 most up-regulated was performed by quantitative PCR (Supplementary Fig. S3A, B). To better understand the roles of the identified genes and their potential interactions, a protein interaction network of the 116 DEGs based on literature was generated. A total of 21 proteins were described as being involved in the same protein complex with at least one other protein. Among the different DEGs, this analysis identified *LGALS3* as the gene with the highest number of known interactions (Fig. 2D).

### Enrichment analysis of the DEGs in the GSDIII^Patients^ vs CTRLs-starved skMt comparison

**Fig. 3:**
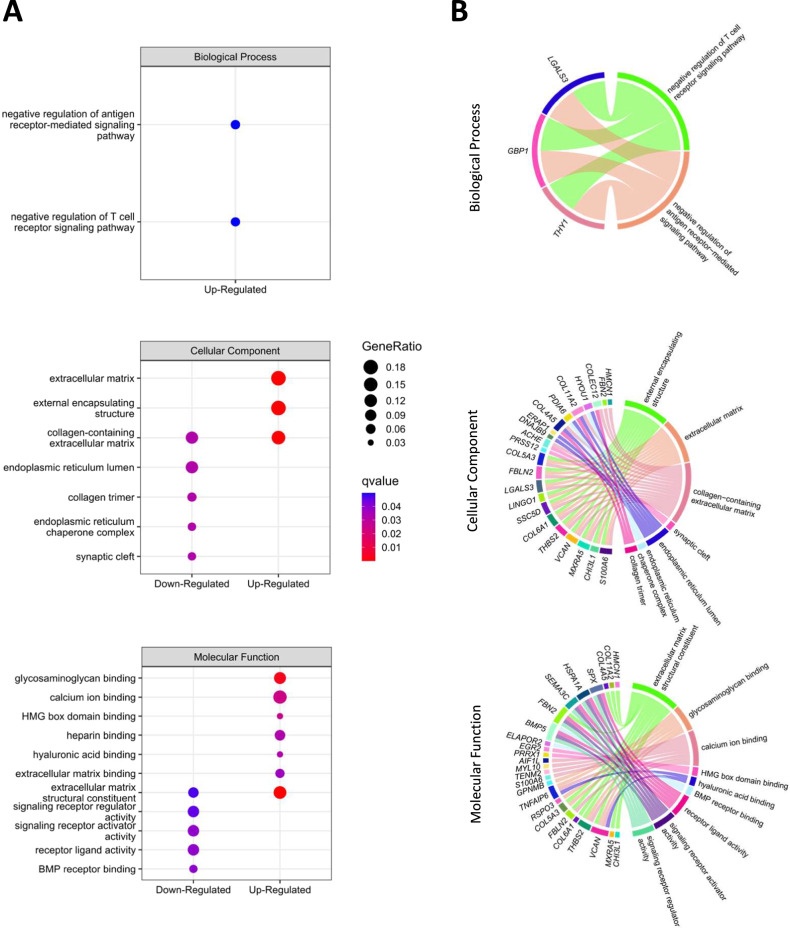
Gene ontology enrichment analysis of the differentially expressed genes in the GSDIII^Patients^ vs CTRLs-starved skMt comparison. **A** Dot plots of enriched GO terms from Biological Process, Cellular Component, and Molecular Function, with a *q* value < 0.05. Dot size represents the Gene Ratio, defined as the ratio of input genes that are annotated in a term, and color gradient represents *q* values. **B** Chord Diagrams of the significant DEGs associated with their enriched GO terms (*q* value < 0.05) for Biological Process, Cellular Component, and Molecular Function. Significant DEGs are shown on the left and enriched GO terms are represented on the right. Left-right connections indicate gene membership in a GO term.

To further investigate the molecular mechanisms at a broader level, an enrichment analysis of the DEGs in the GSDIII^Patients^ compared to the CTRLs-starved skMt was also performed using the Gene Ontology (GO) database [37, 38]. The GO terms are subdivided into three distinct categories that represent different biological aspects: Biological Process, Cellular Component, and Molecular Function. Several GO terms of these three categories were found to be significantly dysregulated. Ranking of up- and down-regulated GO terms was done according to their ratio and enrichment score (Fig. 3A). These included GO terms such as extracellular matrix, external encapsulating structures, collagen-containing extracellular matrix, calcium ion binding, glycosaminoglycan binding, endoplasmic reticulum lumen, signaling receptor regulator activity and receptor-ligand activity. The relationship between genes and GO terms was investigated by chord diagram visualizations (Fig. 3B). Among the genes involved in GO terms, *LGALS3* was identified as being implicated in most of the enriched GO terms suggesting its key role in the muscular pathophysiology of GSDIII.

### Identification of potential GSDIII biomarkers by cross-analysis of RNA sequencing data

Data generated from the bulk RNA sequencing of hiPSC-derived skMt were then crossed with publicly available bulk RNA sequencing data comparing triceps biopsies from *Agl*^+/+^ and *Agl*^−/−^ mouse model (GEO: GSE232166) [27]. As a rescue condition, we also compared the list of DEGs to the data collected from the group of *Agl*^*−/−*^ mice treated with the dual treatment AAV expressing human GDE and rapamycin (GEO: GSE232166) [27]. PCA showed a distinct clustering of the conditions, with *Agl*^*−/−*^ treated mice equidistant from *Agl*^*−/−*^ mice and *Agl*^*+/+*^ mice along PC1 suggesting a potential treatment-induced change in transcriptomic profiles (Supplementary Fig. S4A). *Agl*^*−/−*^ vs *Agl*^*+/+*^ and *Agl*^*−/−*^ treated vs *Agl*^*−/−*^ comparisons were analyzed to respectively identify DEGs in a pathological context and DEGs affected by phenotypically effective treatment. As suggested by the PCA, heatmaps generated on the DEGs of both comparisons also revealed clear clustering of the conditions (Supplementary Fig. S4B, C). In the *Agl*^*−/−*^ vs *Agl*^*+/+*^ comparison, 367 up-regulated genes (372 human) and 87 down-regulated genes (94 human) were identified (Supplementary Fig. S4D). In the *Agl*^*−/−*^ treated vs *Agl*^*−/−*^ comparison, 28 up-regulated genes (29 human) and 161 down-regulated genes (161 human) were identified (Supplementary Fig. S4E).

**Fig. 4:**
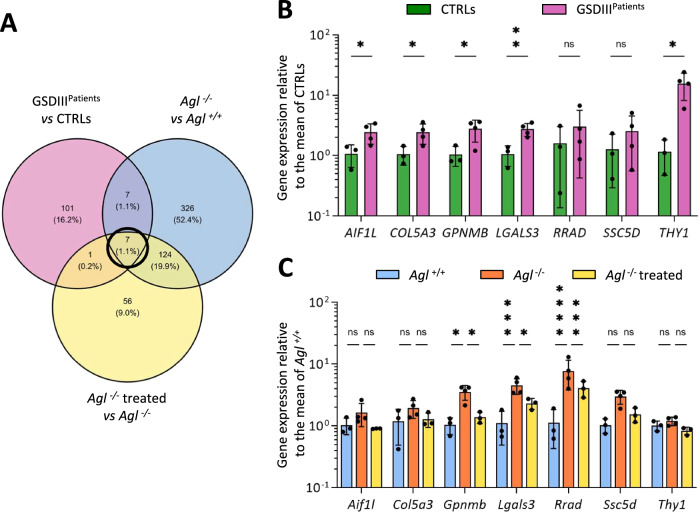
Identification of potential GSDIII biomarkers by cross-analysis of RNAseq data. **A** Venn diagram representing the overlap of DEGs among the three comparisons (GSDIII^Patients^ vs CTRLs, *Agl*^*−/−*^ vs *Agl*^*+/+*^*, Agl*^*−/−*^ treated vs *Agl*^*−/−*^). **B** Gene expression of the seven common dysregulated genes by qPCR in GSDIII^Patients^ and CTRLs-starved skMt. Data are normalized to the mean of CTRLs and are represented as mean ± standard deviation. Statistical analyses were performed using multiple unpaired *t*-tests. *n* = 3 in CTRLs (three dinstint cell lines) and *n* = 4 in GSDIII^Patients^ (four dinstint cell lines). **C** Gene expression of the seven common dysregulated genes by qPCR in *Agl*^*+/+*^*, Agl*^*−/−*^, and *Agl*^*−/−*^ treated mouse triceps biopsies. Data are normalized to the mean of *Agl*^*+/+*^ and are represented as mean ± standard deviation. Statistical analyses were performed using a two-way ANOVA test corrected with the Dunnet method. ****p < 0.0001, ***p < 0.001, **p < 0.01, *p < 0.05. *n* = 3 or 4 mice per condition.

Then, we crossed the human converted DEGs identified in mice triceps biopsies analysis with the DEGs identified in human skMt analysis (Fig. 4A). Interestingly, 7 genes (*AIF1L*, *COL5A3*, *GPNMB*, *LGALS3*, *RRAD*, *SSC5D*, and *THY1*) were identified as commonly differentially expressed in a pathological context in both the human and mouse model. Additionally, these genes were also identified to be re-regulated by the treatment in the mouse model, suggesting they could serve as potential GSDIII biomarkers and therapeutic targets (Fig. 4A). Among them, *GPNMB* and *LGALS3* were confirmed as significantly differentially expressed between all GSDIII^Patients^ and CTRLs-starved skMt (Fig. 4B), between *Agl*^*−/−*^ vs *Agl*^*+/+*^ and between *Agl*^*−/−*^ treated vs *Agl*^*−/−*^ mouse triceps biopsies (Fig. 4C) in quantitative PCR.

### *LGALS3* up-regulation confirmed at the protein level (GAL3) in human skMt, mouse muscle biopsies, and patient muscle biopsies

**Fig. 5:**
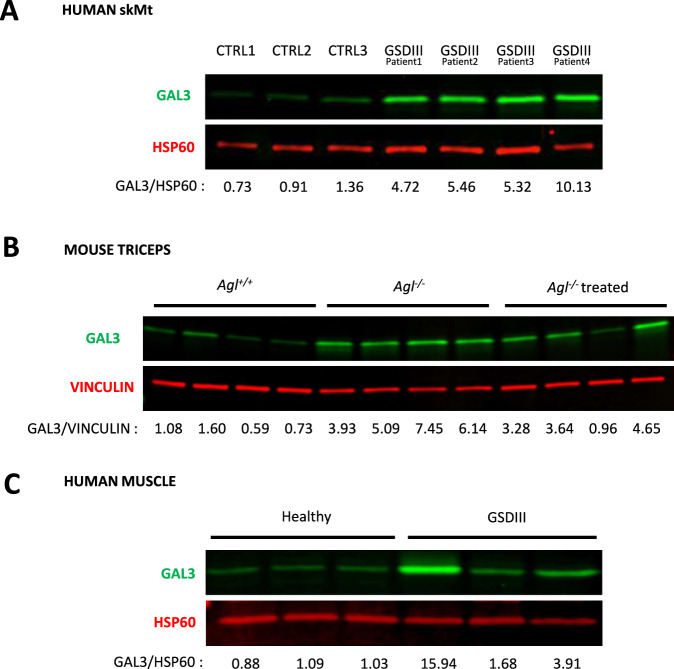
*LGALS3* up-regulation confirmed at the protein level (GAL3) in human skMt, mouse triceps biopsies, and human patient muscle biopsies. Western Blot analysis of GAL3 protein level in respectively CTRLs and GSDIII^Patients^ skMt (**A**), *Agl*^*+/+*^*, Agl*^*−/−*^*, Agl*^*−/−*^ treated mouse triceps biopsies (*n* = 4 independent mice for each condition) (**B**) and healthy and GSDIII patient muscle biopsies (*n* = 3 independent individuals for each condition) (**C**). Quantification of the Western Blot analysis of GAL3 over HSP60 or VINCULIN is normalized respectively to the mean of CTRLs, *Agl*^*+/+*^, and healthy values.

*LGALS3* up-regulation was confirmed at the protein level by GAL3 western blot analysis in the human cell model showing an increase of GAL3 expression in GSDIII^Patients^ skMt compared to CTRLs skMt (Fig. 5A and Original Data 1A). GAL3 western blot analysis was also performed in muscle biopsies of *Agl*^+/+^ mice compared to *Agl*^−/−^ mice and *Agl*^−/−^ treated mice. Results revealed a similar increase of the protein in the mutated group and a decrease in the treated group respectively in triceps (Fig. 5B and Original Data 1B) and quadriceps (Supplementary Fig. S5 and Original Data 2). Finally, to further confirm the up-regulation of GAL3 as a biomarker of GSDIII, western blot analysis was carried out on three muscle biopsies of GSDIII patients described in Laforêt et al. [6]. Although, the GAL3 protein amount is variable, its expression is superior in the three GSDIII patients’ biopsies compared to the three biopsies from healthy individuals, confirming the GAL3 increase in GSDIII patients (Fig. 5C and Original Data 1C). Details concerning human biopsies are described in Supplementary Table S1.

## Discussion

The main result of this study is the identification of GAL3 as a biomarker of the muscle impairments observed in GSDIII. GAL3 is a 29–35 kDa member of the galectin family (β-galactoside binding proteins) characterized by carbohydrate recognition domains. This protein is encoded by the *LGALS3* gene and ubiquitously expressed in adults [39]. It is mainly localized in the cytoplasm but can also be found in the nucleus, at the plasma membrane, or even be secreted [39]. Depending on the cell type and its localization, GAL3 is involved in the regulation of many biological processes such as apoptosis, angiogenesis, inflammation, cell migration, differentiation, preRNA splicing, or fibrosis [39]. GAL3 is commonly considered as a marker of damaged endosomes [40, 41]. It coordinates the removal of vesicles by inducing fusion of lysosomes with autophagosomes through its interactions with TRIM16 [40, 42, 43]. It repairs endosomes by activating the endosomal sorting complexes required for transport machinery [40]. Additionally, it promotes lysosome and autophagosome biogenesis by translocating the transcription factor EB (TFEB) into the nucleus [40, 44].

In the past decade, GAL3 has been found to be dysregulated or considered as a biomarker in various diseases including cardiac [45, 46], renal [45, 47], hepatic [48], cerebrovascular diseases [49], and many types of cancer [45, 50]. While GAL3 was identified as up-regulated in the muscle of mouse model of Duchenne muscular dystrophy [51, 52], amyotrophic lateral sclerosis [53], and merosin-deficient, *lama2*-related congenital muscular dystrophy [52], to date, GAL3 has not been identified as a biomarker in skeletal muscle-related diseases. A very recent study showed the overexpression of *Lgals3* in macrophages extracted from skeletal muscles of mouse models of valosin-containing protein-associated inclusion body myopathy, facioscapulohumeral muscular dystrophy, and Duchenne muscular dystrophy [54]. Authors have also shown the presence of an elevated number of GAL3-positive macrophages in human patient biopsies of Duchenne muscular dystrophy, antisynthetase syndrome, and Limb-girdle muscular dystrophy type R1/2A [54]. Taken together, these studies support our results and seem to indicate that GAL3 is not only a biomarker for GSDIII, but more broadly a potential biomarker of muscle degeneration in myopathies. In addition, we also demonstrated that GAL3 levels are decreased in the rescued mouse condition treated with AAV gene therapy combined with rapamycin. We therefore propose that GAL3 can be considered as a potential biomarker for both diagnosis and the assessment of therapeutic efficacy.

The roles of GAL3 have been extensively studied in the heart, liver, and kidney, as well as in cancer and inflammation [39, 50, 55, 56] but remain unclear in skeletal muscle. To date, only one study investigated GAL3 function in the skeletal muscle context revealing a transient expression of GAL3 in muscle repair [57]. In this study, authors described that muscle repair was compromised in *Lgals3* knock-out mice accompanied by persistent inflammation and the formation of fibrotic tissue at the lesion site [57]. They also showed that GAL3 participates in the differentiation process of myoblasts into myotubes in in vitro primary mouse cells, confirming previous results on C2C12 cells [58]. In GSDIII patient biopsies, glycogen accumulates either in the intermyofibrillar space disrupting myofibrillar architecture, or in autophagosomes associated with increased autophagic flux [6]. Given this observation and the known role of GAL3 in lysosomal damage, we hypothesize an activation of lysosomal glycogen degradation as a compensatory mechanism. This hypothesis is consistent with the fact that the only other gene significantly up-regulated in both mouse triceps biopsies and human skMt is melanoma-associated transmembrane protein (GPNMB). Indeed, GPNMB has been previously described as being localized on autophagosomes allowing the recruitment of LC3 to induce the fusion with lysosomes. More broadly GPNMB has been described as essential for tissue repair [59]. Based on our results and these studies, it is probable that the up-regulation of GAL3 may reflect damaged autolysosomes overloaded with glycogen in an attempt to send cytosolic glycogen through the lysosomal degradation pathway as a compensatory mechanism. Further experiments are needed to explore this hypothesis and the precise role of GAL3 in the molecular mechanisms underlying the skeletal muscle physiopathology of GSDIII and more in general in other neuromuscular diseases.

Because of the central role of GAL3 in various biological pathways, GAL3 is already a therapeutic target in several fibrotic and inflammatory diseases through the use of inhibitory molecules [48, 60, 61]. Our compensatory mechanism hypothesis would suggest that GAL3 activation or overexpression should be considered as a potential therapeutic approach for GSDIII rather than its inhibition. Molecularly, GAL3 orchestrates the replacement of damaged lysosomes by inactivating mTOR which leads to the dephosphorylation of TFEB that is then sent to the nucleus to activate lysosome biogenesis [40, 44]. As GAL3, TFEB has also been shown to promote lysosome exocytosis and induce cellular clearance [62]. Recently, AAV-overexpressing TFEB has been evaluated in several models of Pompe disease resulting in an increased formation and exocytosis of autolysosomes [63]. In 2013, another study reported a reduction of glycogen accumulation and an increase of GAA activity in skeletal muscle derived from Pompe’s hiPSC using a lentiviral vector overexpressing TFEB, suggesting the instrumental use of autophagy induction to reduce glycogen content [64]. In 2017, systemic delivery AAV-mediated overexpression of TFEB was evaluated in the Pompe mouse model showing a statistically significant improvement in muscular performance of treated animals compared to untreated mice while total glycogen content remained unchanged [65]. Although these results may suggest a discrepancy with previous studies, authors discussed the potential other metabolic effects of TFEB and the low levels of infection in the muscles to explain the lack of changes in glycogen levels in tissues from treated animals. In GSDIII, a very recent study demonstrated that activation of autophagy by rapamycin treatment (targeted mTOR) was not sufficient to clear glycogen in the muscle of GSDIII mouse but when used in combination with gene therapy it acted in synergism [27]. These data suggest that activating autophagy, by targeting different key players such as TFEB or mTOR, is a promising approach to treat the muscle phenotype of GSDIII, especially for combination treatment. Following the same paradigm, given its known role in the regulation of TFEB and autophagy, GAL3 could also be a potential target to activate autophagy in GSDIII skeletal muscles.

In conclusion, our work has highlighted the value of cross-referencing sequencing data from two distinct pathological models, both animal and cellular, to identify a new GSDIII biomarker. *LGALS3* gene, encoding the GAL3 protein, has been demonstrated as significantly overexpressed in disease conditions, and this overexpression was further confirmed in muscle biopsies from GSDIII patients, strengthening the robustness of our findings. Additionally, we demonstrated that GAL3 levels were regulated in mice treated with an ongoing developing therapy, highlighting the biomarker’s pharmacodynamic potential.

Our study confirmed the predictive value of both GSDIII models used, particularly the recently developed cellular model [33]. As this model can be miniaturized, it opens the possibility of large-scale screening, which should accelerate therapeutic development for GSDIII. While GAL3 is a promising biomarker for therapeutic evaluation, its current application for diagnosing GSDIII is limited due to the invasive nature of muscle biopsies, which are not commonly performed on GSDIII patients. To broaden its diagnostic potential, detecting GAL3 in serum samples of GSDIII patients would be highly beneficial, allowing for non-invasive monitoring in clinical settings. Further research is required to explore the feasibility of such an approach and to evaluate the wider clinical application of GAL3 for GSDIII.

## Materials and methods

### hiPSC lines

CTRL1 hiPSC line is a commercial cell line reprogrammed from healthy peripheral blood mononuclear cells (PC1426, newly referenced PCi_CAU2) provided by Phenocell (Grasse, France). CTRL2 hiPSC line was reprogrammed with the Sendaï virus technique at IStem from healthy fibroblast (GM1869) provided by Coriell Institute (Camden, NJ, United States) [66] and modified to express an inducible Cas9 in AAVS1 locus. CTRL3 hiPSC line was reprogrammed with episome technique at IStem from healthy IMR-90 lung fibroblasts (CCL-186) [67] provided by ATCC Cell Lines Biology Collection (CCL-186, Washington, DC, United States). GSDIII^Patient1^ hiPSC line was reprogrammed with episome technique by Phenocell (Grasse, France) from GSDIII patient fibroblasts (GM0576) by Coriell Institute (Camden, NJ, United States) [33]. GSDIII^Patient2^, GSDIII^Patient3^, and GSDIII^Patient4^ hiPSC lines were reprogrammed with the Sendaï virus technique at IStem from GSDIII patient fibroblasts (GM3390, GM0303, GM2523) provided by Coriell Institute (Camden, NJ, United States) [34].

### Cell culture and differentiation

hiPSC were cultured on matrigel (#254277, Corning, United States) or vitronectin (#A14700, Gibco, United States)-coated culture dishes with iPS-Brew XF medium (#130-1043-68, Miltenyi Biotec, Germany) renewed every 2 days. They were passaged every 5–7 days using a single-cell method with StemPro Accutase Cell dissociation agent (#A1110501, ThermoFisher Scientific, United States) and the addition of Y-27632 Rock inhibitor (#130-104-169, Miltenyi Biotec, Germany) for 24 h after replating.

hiPSCs were differentiated into skMt using a method developed by Geneabiocell® [35]. Seeding of hiPSCs was performed on collagen I-coated plates (#354400, Corning, United States) and hiPSCs were maintained for 10 days in skeletal muscle induction medium (#SKM01, AMSBIO, United Kingdom) with a passage on day 7. Dissociation of the cells was then performed using 0.05% trypsin (#25300054, ThermoFisher Scientific, United States) and seeding onto collagen I-coated plates for 7 days in skeletal myoblast medium (#SKM02, AMSBIO, United Kingdom) until frozen. SkMb were thawed on collagen I-coated plates in SKM02 medium until confluency. They were then incubated with a skeletal muscle differentiation medium (#SKM03, AMSBIO, United Kingdom) for 3 additional days. For glucose starvation experiments, skMt were incubated with glucose-free DMEM (#11966-025, ThermoFisher Scientific, United States) supplemented with 10% Fetal Bovine Serum (#F7524, Sigma-Aldrich, United States) for further 2.5 or 5 days.

### Human muscle patient biopsies

This study was approved and performed under the ethical guidelines issued by the different involved institutions and in compliance with the Helsinki Declaration. Informed consent was obtained from all patients. GSDIII patient muscle biopsies belong to P9, P6, and P3 described in Laforêt et al. [6].

### RNA extraction

Human skMt was lysed with RLT Plus buffer (#1030963, Qiagen, Germany). Mouse triceps biopsies were lysed with bead lysis tubes (#740814.50, Macherey-Nagel, Germany) using Qiazol (#79306, Qiagen, The Netherlands) and chloroform (#102444, Sigma-Aldrich, United States) reagents. Total RNA was isolated using an RNeasy Plus Mini extraction kit (#74134, Qiagen, Germany) according to the manufacturer’s instructions. DNase I digestion (#18047019, Invitrogen, United States) was performed to degrade DNA in the sample. RNA levels and quality were checked using the NanoDrop® spectrophotometer (ThermoFisher Scientific, United States).

### Reverse transcription and quantitative PCR

A total of 500 ng of RNA was reverse transcribed using the SuperScript III reverse transcription kit (#18080085, Invitrogen, United States). Quantitative polymerase chain reaction (qPCR) was conducted using a QuantStudio 12 K Flex real-time PCR system (Applied biosystem, United States) and Luminaris Color HiGreen qPCR Master Mix (#K0973, Thermo Scientific, United States), according to the manufacturer’s guidelines. Quantification of gene expression was based on the Delta Ct method, normalized on 18S (human) or P0 (mouse) gene expression, and normalized to the appropriate group value (CTRLs, *Agl*^*+/+*^, or Day 0). The primers used in this study are reported in Supplementary Table S2.

### Bulk RNA sequencing

Quantification and quality control of RNA samples were determined using the Agilent RNA ScreenTape System (#5067-5576, Agilent, United States) according to the manufacturer’s guidelines. The RNA Integrity Number of all samples was superior to 9. The QuantSeq 3′ mRNA-Seq Library Kit FWD from Illumina was used to generate sequencing libraries. Briefly, library generation was started from 100 ng of mRNA by oligo(dT) priming and first-strand synthesis. Random primers initiated the second strand synthesis, and the final libraries were PCR amplified and barcoded in 17 cycles. All libraries were quantified using the Agilent High Sensitivity DNA Kit and pooled for sequencing. 2 nM of pooled libraries were denatured, and a quantity of 1.8 pmol was used for cluster generation before single-end sequencing on an Illumina NextSeq 550 (High Output 1 × 75 bp run). Samples were sequenced with an average of 19,113,179 reads (single‐end) per sample. The quality control of the sequencing data was evaluated using FastQC (v0.11.9). The reads were trimmed using Prinseq-lite (v0.20.4) [68] (‐‐trim‐right 20) and filtered by average quality score (‐‐trim‐qual 20) and cutadapt (v4.1) [69]. Reads were mapped EnsEMBL GRCh37.87 human reference using rna‐STAR (v2.7.6a) [70]. Reads below a mapping score of 10 or multimapped were filtered using samtools (v0.1.13) [71]. The gene expression level in each sample was quantified with HTSeq‐count (v0.99.2, Python3.10) [72]. The differential gene expression between conditions was calculated with DESeq2 (v1.34.0 using R v4.1.2) [73, 74]. Genes were considered differentially expressed when their BaseMean is greater than 40, their adjusted *p* value is less than 0.05, and |log2FoldChange| is upper than 2.

### Transcriptomic analysis

All following analyses were based on the expression data from GSDIII^patients^ vs CTRLs-starved skMt. PCA was conducted using Plotly on the mRNA expression data, employing a variance stabilizing transformation (vst function) from the DESeq2 package [74, 75]. A heatmap of the DEGs was generated using the ComplexHeatmap package on the mRNA expression data also using the variance stabilizing transformation (vst function) from the DESeq2 package [76, 77]. A volcano plot of DEGs was constructed using the EnhancedVolcano R package (https://www.r-project.org). The STRING database version 12.0 was employed to generate the protein–protein interaction network based on all DEGs [78]. Parameters included a physical subnetwork type and a minimum interaction score of 0.4. Disconnected nodes were excluded from the network. Functional annotation analysis of DEGs was conducted using the clusterProfiler R package [79, 80]. The GO database and its subcategories known as Biological Process, Molecular Function, and Cellular Component, were used to identify enriched GO terms among the DEGs. A GO term was considered significant if associated with an adjusted *q* value of less than 0.05 (http://bioconductor.org/packages/qvalue). To perform the cross-analysis of the human DEGs of GSDIII^patients^ vs CTRLs-starved skMt and the DEGs obtained from *Agl*^*−/−*^ vs *Agl*^*+/+*^ and *Agl*^*−/−*^ treated vs *Agl*^*−/−*^ in mice, we converted the mouse gene symbols (MGI) into human gene symbols (HGNC). This conversion resulted in a many-to-many relationship between mouse and human genes. We retained only the DEGs from the mouse data that corresponded to a human gene symbol. A Venn diagram was then generated from the human-based DEGs of the three datasets using the ggvenn R package (https://cran.r-project.org/web/packages/ggvenn/index.html).

### Immunostaining

Fixation of skMt was performed using 4% paraformaldehyde (#15710, Euromedex, France) for 10 min at room temperature. After three washes in phosphate-buffered saline (PBS), permeabilization of cells was performed using 0.5% Triton X-100 (#93443, Sigma, United States) for 10 min and blocking using PBS solution supplemented with 1% bovine serum albumin (#A9647, Sigma, United States) for 1 h at room temperature. Staining of the cells was then performed with primary antibodies (listed in Supplementary Table S3) incubated overnight at 4 °C. After three washes in PBS, staining was revealed by appropriate Alexa Fluor secondary antibodies (listed in Supplementary Table S3) incubated for 1 h at room temperature, and nuclei were visualized with Hoechst solution 1: 2000 (#H3570, Invitrogen, United States). Imaging has been performed with an HCS Navigator™ (Version 6.6.0) software-associated Cell Insight™ CX7 Platform automated microscope (ThermoFisher Scientific, United States) with a ×20 objective.

### Protein extraction and western blot analysis

Human skMt proteins were extracted with NP40 lysis buffer (#FNN021, Thermo Scientific, United States) supplemented with 1X protease and phosphatase inhibitor cocktail (#1811284, Thermo Scientific, United States). Mouse triceps and quadriceps biopsies and human biopsies were lysed with bead lysis tubes (#740814.50, Macherey-Nagel, Germany) in RIPA buffer (#R0278, Sigma-Aldrich, United States) supplemented with 1X Complete™ protease-inhibitor cocktail (#11836153001, Sigma-Aldrich, United States). Cell and tissue lysates were then centrifuged at 5 °C and 12,000 × *g* for 15 min and the supernatant was collected. Human biopsy tissue protein supernatants were concentrated using the Amicon® Ultra-0.5 centrifugal filter device with a 10 kDa molecular weight cut-off filter (#UFC5010, Merck Millipore, Germany) following the manufacturer’s instructions. Protein concentration was determined using the Pierce BCA Protein Assay Kit (#23227, Thermo Scientific, United States) and absorbance was read at 562 nm with a CLARIOstar® microplate reader (BMG Labtech, Germany). For western blot analysis, a total of 30 μg of protein for mice muscle biopsies and human skMt and 15 μg of protein for human patient biopsies was separated using a 4%–15% Criterion™ XT tris-glycine protein gel (#5671083, BioRad, United States) and then transferred on PVDF membrane (#1704157, BioRad, United States) with a Trans-Blot Turbo Transfert system (BioRad, United States) according to the manufacturer’s guidelines. Membrane blocking was performed using Odyssey blocking buffer (#927-70001, Li-Cor, United States) for 1 h at room temperature and incubation with primary antibodies (listed in Supplementary Table S3) diluted in blocking buffer was performed at 4 °C overnight or at room temperature for 2 h. After three washes of 10 min at room temperature with TBS + 0.1% Tween 20 (#28829.296, VWR, United States), incubation of the membrane was performed with appropriate fluorescent secondary antibodies (listed in Supplementary Table S3) in blocking buffer at room temperature for 2 h. After three washes, proteins were revealed using Odyssey® Clx (Li-Cor, United States) according to the manufacturer’s guidelines. VINCULIN was used as a loading control in mouse samples. HSP60 was used as a loading control in human samples and was detected at 60 kDa in human skMt samples and 40 kDa in human patient biopsies samples.

### Glycogen staining by periodic acid Schiff staining

PAS staining was performed with the PAS Staining Kit (#395B, Sigma-Aldrich, United States) according to the manufacturer’s guidelines. Briefly, fixation of the cells was performed with 4% paraformaldehyde for 10 min at room temperature and then washed in PBS. Periodic Acid solution was then added on cells for 5 min at room temperature. After three washes in tap water, Schiff’s reagent was then added on cells for 15 min at room temperature. After four final washes in tap water, staining was analyzed using an EVOS XL Core microscope (Invitrogen, United States).

### Glycogen content measurement by indirect enzymatic test

Glycogen content of skMt was measured using the Glycogen-Glo assay (#J5052, Promega, United States) according to the manufacturer’s guidelines. Cells were lysed using NP40 lysis buffer (#J60766.AK, ThermoFisher Scientific, United States). To induce glycogen digestion into glucose, lysates were incubated with the glucoamylase enzyme for 1 h at room temperature. The generated glucose was then measured using glucose dehydrogenase in combination with a bioluminescent NADH detection reagent which was incubated for 1.5 h at room temperature. The resulting light signal was proportional to the initial glycogen concentration in skMt. Luminescence was quantified using a CLARIOstar® microplate reader (BMG Labtech, Germany). Raw data corresponding to glycogen content were normalized on the number of viable cells quantified by luminescence with CellTiter-Glo assay (#G9242, Promega, United States).

### Statistical analysis

Data are represented as mean ± standard deviation. GraphPad Prism 7.0 software (GraphPad Software, United States) was used for statistical analyses. *p* value < 0.05 or adjusted *p* value < 0.05 were considered significant. Unpaired *t*-tests or multiple unpaired *t*-tests were performed for two groups analysis and two-way ANOVA tests corrected with the Dunnet method were performed for three groups analysis. All statistical tests were performed two-sided. The statistical analysis performed for each dataset is indicated in the figure legends.

## Supplementary information

Revised Supplementary Figures

Revised Original Data—Uncropped Western Blot

## Data Availability

All source data used in this article are available on GEO under the accession codes GEO: GSE272786, and GSE232166, respectively for the new bulk and for the publicly available RNAseq data. All relevant scripts are available on GitHub: https://github.com/I-Stem-CECS/NGS82. The datasets used and/or analyzed during the present study are also available from the corresponding author on reasonable request.
