# Supplementary material for: Galectin-3: a novel biomarker of glycogen storage disease type III
Source: Cell Death Discov. 2025 Apr 14;11:173. doi: 10.1038/s41420-025-02452-6 (PMC11997124; doi:10.1038/s41420-025-02452-6)
Supplement: Supplementary file 1 — Revised Supplementary Figures [file 41420_2025_2452_MOESM1_ESM.pdf]

Supplemental Figure S1

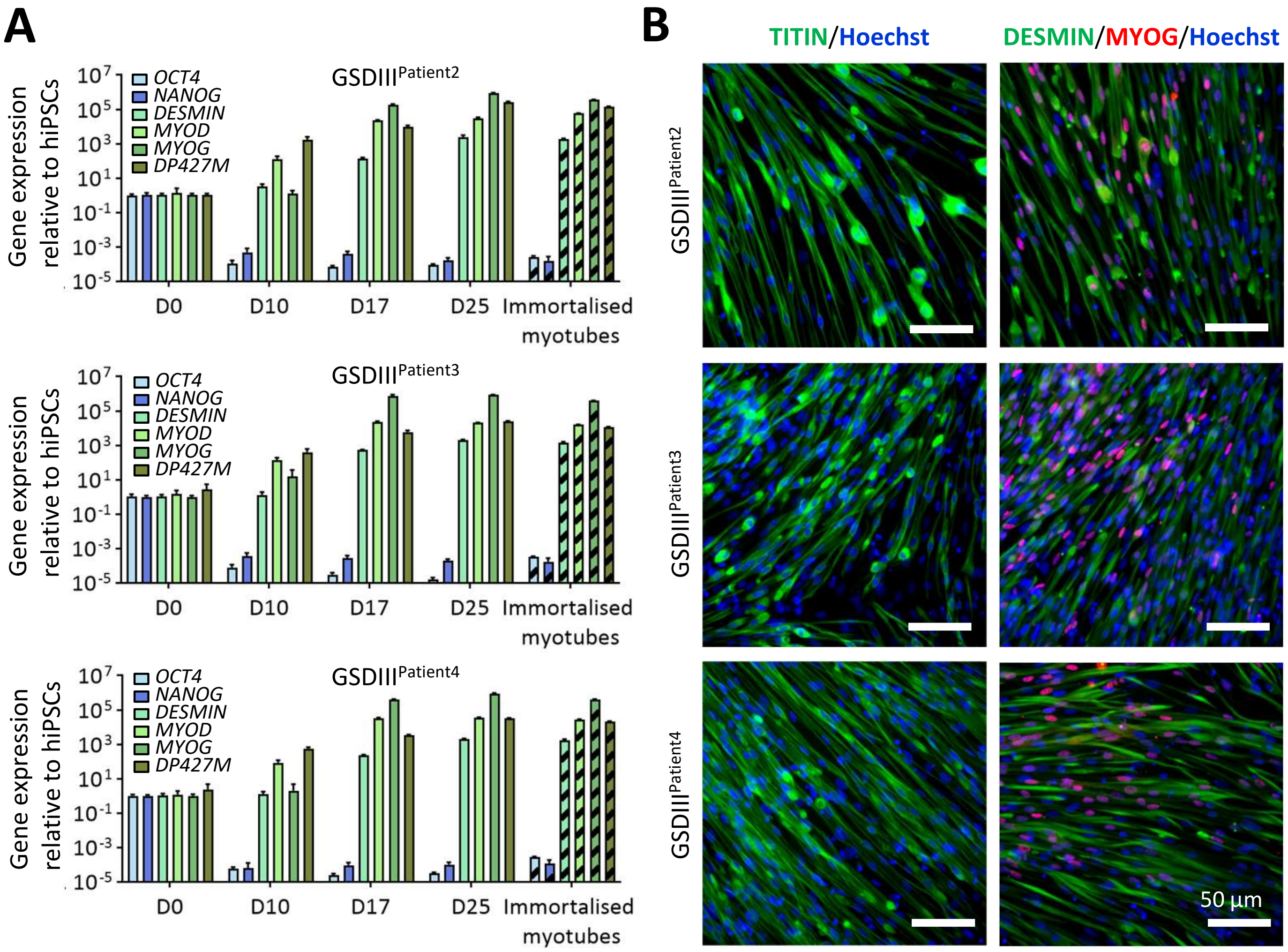

Supplemental Figure S1. Skeletal myogenic differentiation of GSDIII^{Patient2}, GSDIII^{Patient3} and GSDIII^{Patient4} skMt. (**A**) mRNA levels of pluripotency markers (*OCT4, NANOG*) and myogenic markers (*DESMIN, MYOD, MYOG, DP427M*) measured by qPCR in triplicate at day 0 (hiPSCs), day 10 (myogenic precursor), day 17 (skMb) and day 25 (skMt) of differentiation of GSDIII^{Patient2}, GSDIII^{Patient3} and GSDIII^{Patient4} cell lines. mRNA levels are normalised to hiPSCs at day 0. Immortalised myotubes are used as a control of the gene expression profile of terminated differentiation. (**B**) TITIN (green), DESMIN (green) and MYOG (red) staining by immunofluorescence in GSDIII^{Patient2}, GSDIII^{Patient3} and GSDIII^{Patient4} skMt. Nuclei are labelled by Hoechst staining (blue). Scale bar = 50 µm.

Supplemental Figure S2

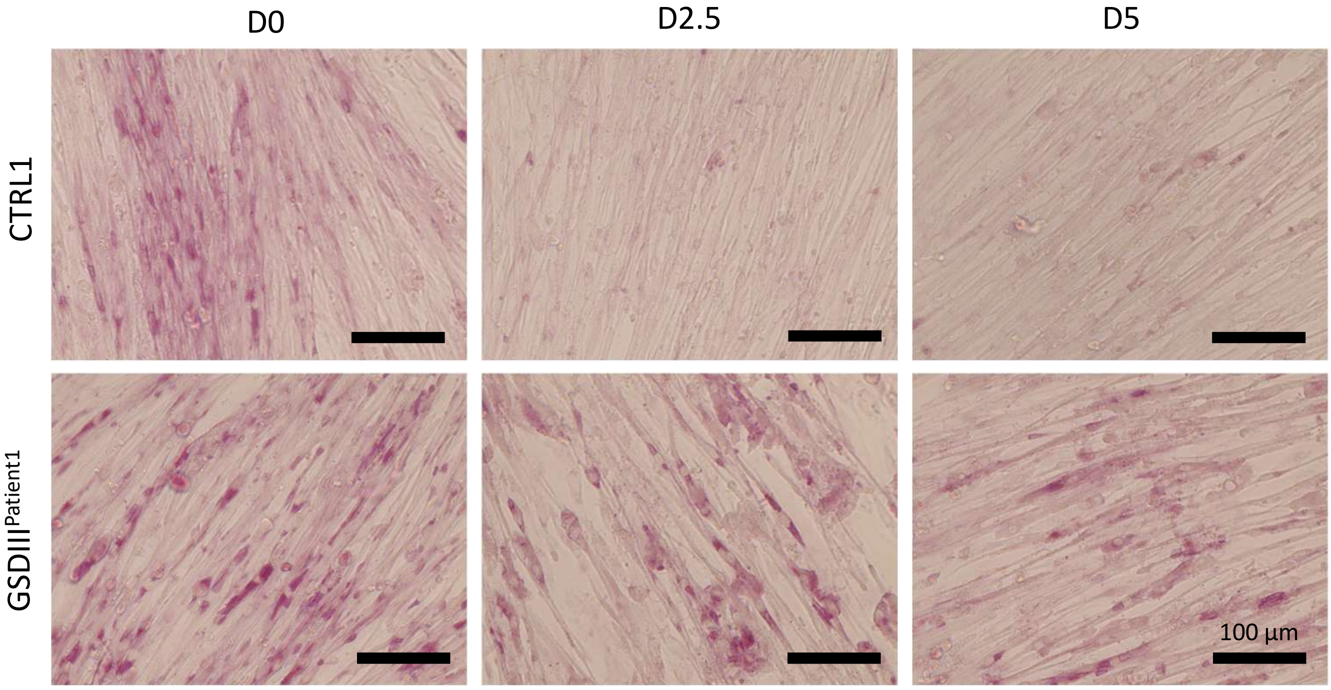

Supplemental Figure S2. Glycogen consumption along starvation time in GSDIII^{Patient1} and CTRL1 skMt. Periodic acid Sciff staining performed at day 0 (D0), day 2.5 (D2.5) and day 5 (D5) of starvation time in GSDIII^{Patient1} and CTRL1 skMt. Scale bar = 100 µm.

Supplemental Figure S3

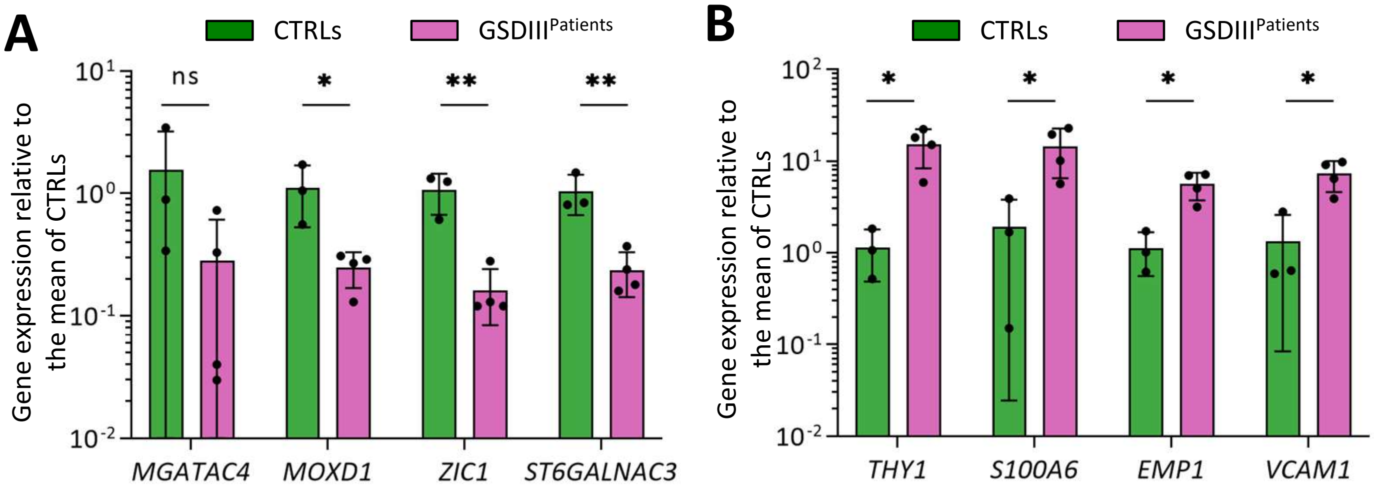

Supplemental Figure S3. Validation of the most dysregulated genes in bulk RNAseq analysis of GSDIII^{Patient} vs CTRL starved skMt. (**A**) Gene expression of the 4 most up-regulated genes by qPCR. (**B**) Gene expression of the 4 most down-regulated genes by qPCR. (**A,B**) Data are normalised to the mean of CTRLs and are represented as mean ± standard deviation. Statistical analyses were performed using multiple unpaired t-test. $p < 0.0001$: ****, $p < 0.001$: ***, $p < 0.01$: **, $p < 0.05$: *. n=3 in CTRLs (3 distinct cell lines) and n=4 in GSDIII^{Patients} (4 distinct cell lines).

Supplemental Figure S4

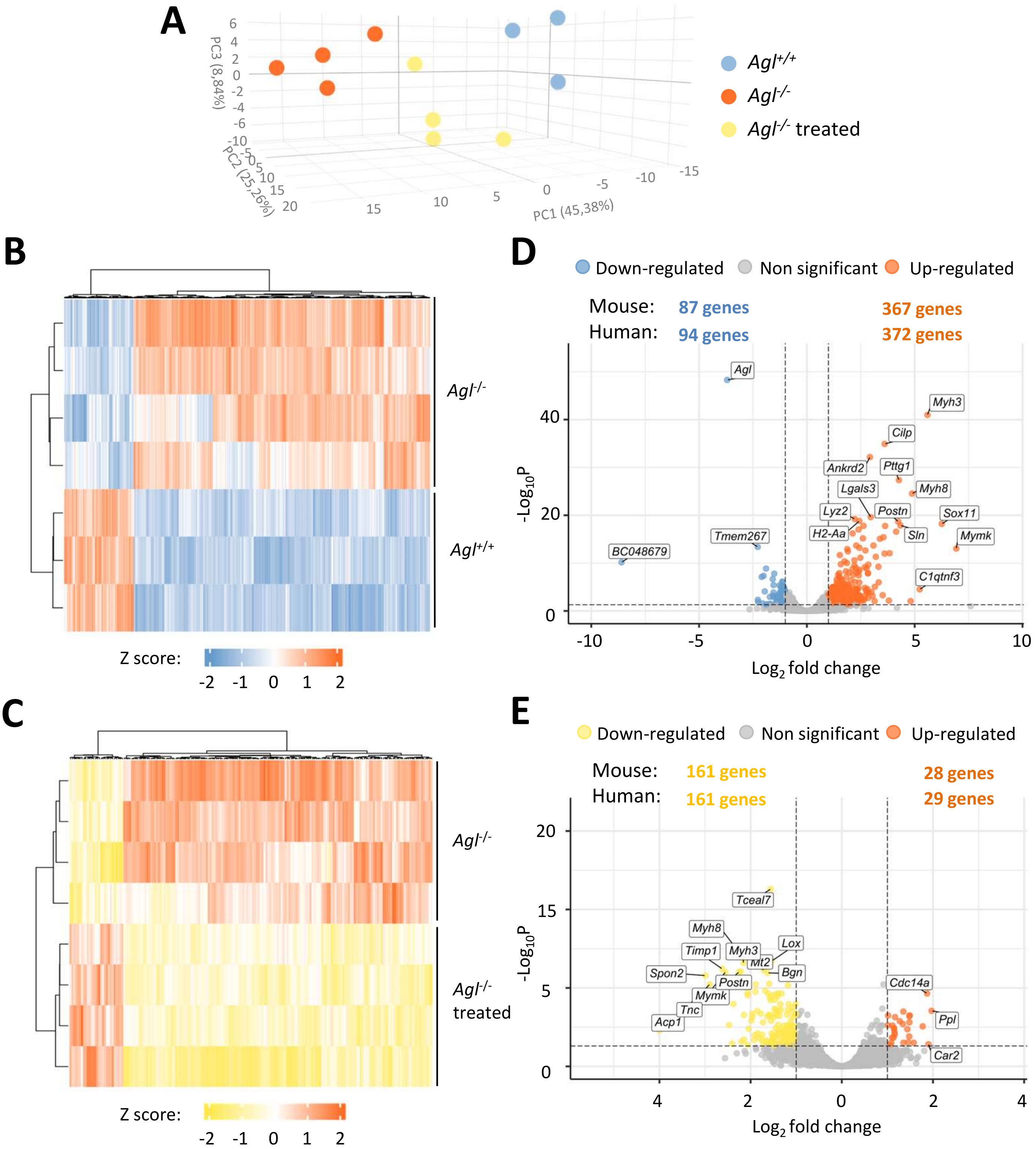

Supplemental Figure S4. Bulk RNAseq analysis of *Agl-/-* vs *Agl+/+* and *Agl-/-* treated vs *Agl-/-* mouse triceps biopsies. (**A**) Principal Component Analysis (PCA) representing the variability of the 3 conditions. n=3 *Agl+/+ mice,* n=4 *Agl-/- mice,* n=4 *Agl-/-* treated mice. (**B,C**) Heatmap representation of HCA showing clustering of detailed conditions based on DEGs of *Agl-/-* vs *Agl+/+* and *Agl-/-* treated vs *Agl-/-* respectively. (**D,E**) Volcano plot representation of down (blue, yellow) and up (orange) DEGs of *Agl-/-* vs *Agl+/+* and *Agl-/-* treated *vs Agl-/-* respectively. The number of DEGs corresponding to mouse gene list or human corresponding gene list are mentioned for both comparisons. Corrected p-value < 0.05. |fold change| > 2.

Supplemental Figure S5

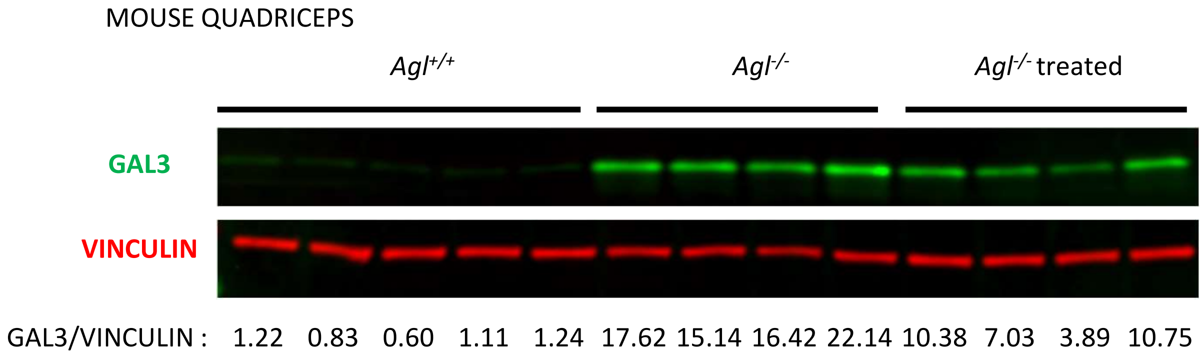

Supplemental Figure S5. *LGALS3* up-regulation confirmed at the protein level (GAL3) in mouse quadriceps biopsies. Western Blot analysis of GAL3 protein level in $Agl^{+/+}$, $Agl^{-/-}$, $Agl^{-/-}$ treated mouse triceps biopsies. (n=4 or 5 independent mice for each condition). Quantification of the Western Blot analysis of GAL3 over VINCULIN are normalised to the mean of $Agl^{+/+}$ values.

Supplemental Table S1

Human biopsies details			
**Age at biopsy**	**Sexe**	**Sampled Muscles**	**Condition**
43	M	Quadriceps	CTRL
23	M	Deltoide	CTRL
38	M	Deltoide	CTRL
53	M	Quadriceps	GSDIII
24	M	Deltoide	GSDIII
40	M	Deltoide	GSDIII

Supplemental Table S1. Details of human patient biopsies.

Supplemental Table S2

Gene	Forward (5'→3')	Reverse (5'→3')
Human		
18S	TCTTCAGTCCGCTCCAGGTCT	GAGGATGAGGTGGAACGTGT
OCT4	CCTCACTTCACTGCACTGTA	CAGGTTTTCTTTCCCTAGCT
NANOG	CAAAGGCAAACAACCCACTT	TCTGCTGGAGGCTGAGGTAT
DESMIN	ATTGGAGGACCGATTTGCC	TCACCGTCTTCTTGGTATGGA
MYOD	GGGGCTAGGTTCAGCTTTCT	CTACATTTGGGACCGGAGTG
MYOG	TAAGGTGTGTAAGAGGAAGTCG	CCACAGACACATCTTCCACTGT
DP427m	GTGGGAAGAAGTAGAGGACTGT	TCCTGTAGGTCACTGAAGAGGT
MGATAC4	GGGGAAACCACCTTCAACAGGAG	CGTTTTCCCCAACATCTAGGGCT
MOXD1	GCACTTTGGAGTGCCTGGAAGA	AATGACGCAGCCTGATGCCTCT
ZIC1	GATGTGCGACAAGTCCTACACG	TGGAGGATTCGTAGCCAGAGCT
ST6GALNAC3	TACGTGACCACAGAGAAGCGCA	CGTGAATGCCATAACAGGCGTC
S100A6	AAGCTGCAGGATGCTGAAAT	CCCTTGAGGGCTTCATTGTA
EMP1	ATGCCAGTGAAGATGCCCTC	TGTAGATGGACACCCCCACA
VCAM1	GATTCTGTGCCCACAGTAAGGC	TGGTCACAGAGCCACCTTCTTG
AiF1L	CCTTCCAGAAAAGCTCACAGCC	CTTCATCTCCAGGTGGGTCTTG
Col5a3	TGACCGGGCATTCAGAATTGG	CGGGCACCCCTTTCATCAT
GPNMB	GTGCTCAATGGAACCTTCAGCC	AGGAATCCTACTCAGCTCCAGG
LGALS3	GTGCCTCGCATGCTGATAAC	TGTTTGCATTGGGCTTCACC
RRAD	CGAGAGCGTTTACAAGGTGCTG	ATGGAGCGATCATAGGTGTGCC
SSC5D	TGTGACTGCCAGTGTTCTGGAG	GGAGTGTTTCGTGGTTGGCATC
THY1	GAAGGTCCTCTACTTATCCGCC	TGATGCCCTCACACTTGACCAG
Mouse		
P0	CTCCAAGCAGATGCAGCACA	ATAGCCTTGCGCATCATGGT
Aif1l	ATGTCTTACCCGAGTGGGGA	GAAATGGGGGCAGAGATGCT
Col5a3	GGCAAAGATGGTATTCCAGGACC	TGCTTCCTTTGTGACCAGGCATC
Gpnmb	AGGATCCATTGCTCCAGGAC	CTGCACAGCTCACATACATGC
Lgals3	AACACGAAGCAGGACAATAACTGG	GCAGTAGGTGAGCATCGTTGAC
Rrad	TCTCGTGAGGTCTCTGTGGATG	CCTCGAATAGTGCCTGGACATTG
Ssc5d	AGGCTTCACTGTCAGAATGCCC	GCTCCAACTGAAAGGGTCTGAG
Thy1	CCTTACCCTAGCCAACTTCACC	TTATGCCGCCACACTTGACCAG

Supplemental Table S2. List of primer sequences used for quantitative PCR.

Supplemental Table S3

Antibody	Supplier	Reference	dilution
Western Blot			
GDE	Agrisera	AS09 454	1:1000
HSP60	Santa Cruz	Sc-59567	1:200
GAL3	R&D Systems	AF1197-SP	1:1000
VINCULIN	Abcam	129002	1:1000
IRDye® 680RD Donkey anti-Goat IgG	Li-Cor	926-68074	1:5000
IRDye® 800CW Donkey anti-Rabbit	Li-Cor	926-32213	1:10000
IRDye® 800CW Donkey anti-Mouse	Li-Cor	926-32212	1:10000
Immunofluorescence			
DESMIN	R&D Systems	AF3844	1:200
MYOG	DSHB	F5D-c	1:50
TITIN	US Biological	T5650	1:50
Donkey anti-mouse AF488	Invitrogen	A-21202	1:1000
Donkey anti-goat AF488	Invitrogen	A-11055	1:1000
Donkey anti-mouse AF555	Invitrogen	A-31570	1:1000

Supplemental Table S3. List of antibody references and dilutions used in Western Blot and immunofluorescence techniques.
