# Supplementary material for: Galectin-3: a novel biomarker of glycogen storage disease type III
Source: Cell Death Discov. 2025 Apr 14;11:173. doi: 10.1038/s41420-025-02452-6 (PMC11997124; doi:10.1038/s41420-025-02452-6)
Supplement: Supplementary file 2 — Revised Original Data—Uncropped Western Blot [file 41420_2025_2452_MOESM2_ESM.pdf]

Original Data 1

A

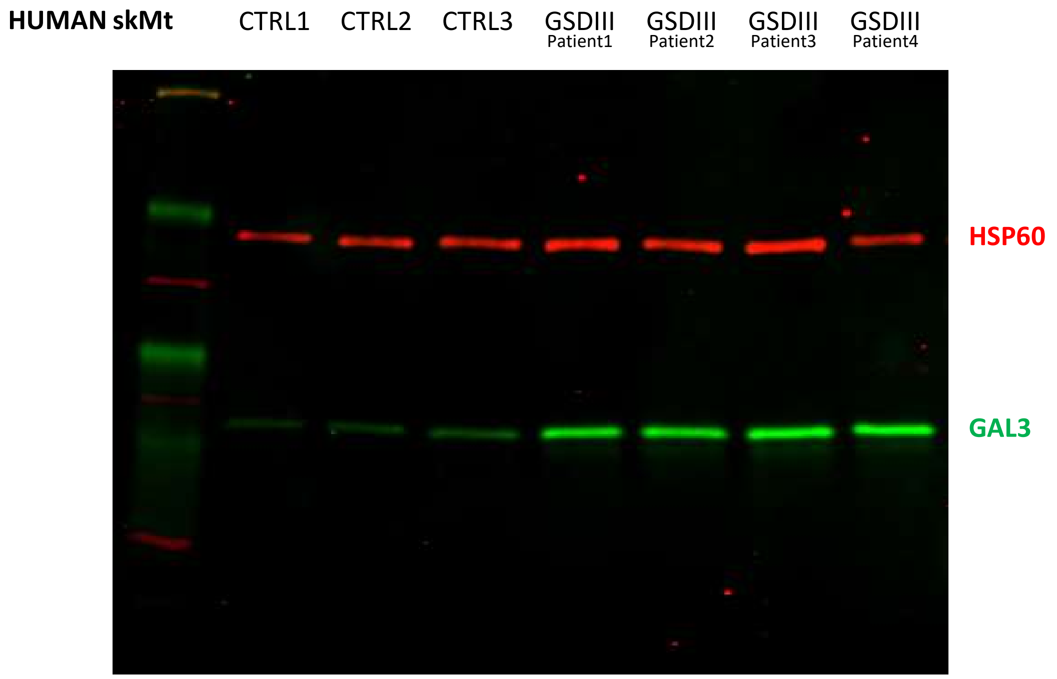

B

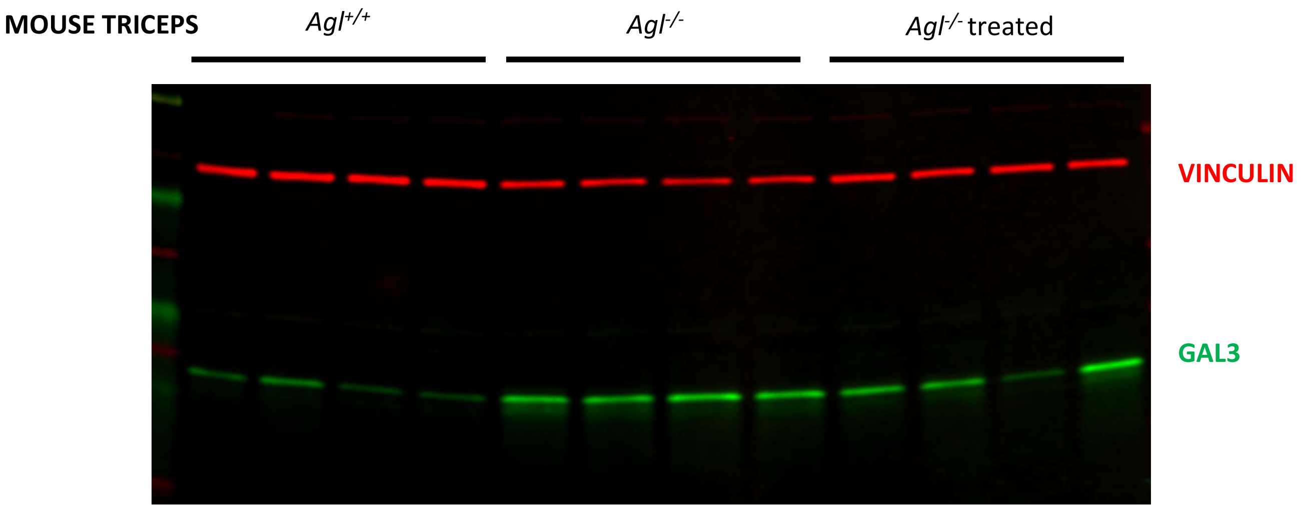

C

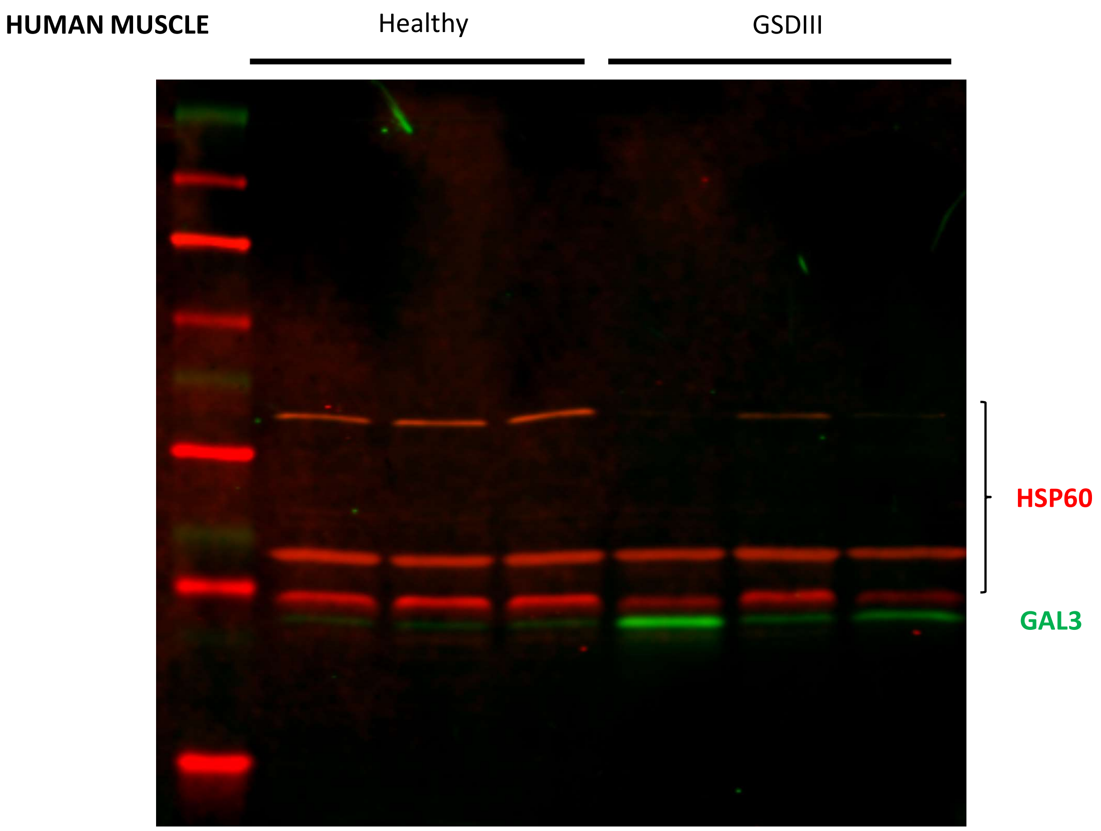

Original Data 1. *LGALS3* up regulation confirmed at the protein level (GAL3) in human skMt, mouse triceps biopsies and human patient muscle biopsies. (A,B,C) Uncropped Western Blot analysis of GAL3 protein level in respectively CTRLs and $GSDIII^{Patients}$ skMt (A) , $Agl^{+/+}$, $Agl^{-/-}$, $Agl^{-/-}$ treated mouse triceps biopsies (n=4 independant mice for each condition) (B) and healthy and GSDIII patient muscle biopsies (n=3 independant individuals for each condition) (C).

Original Data 2

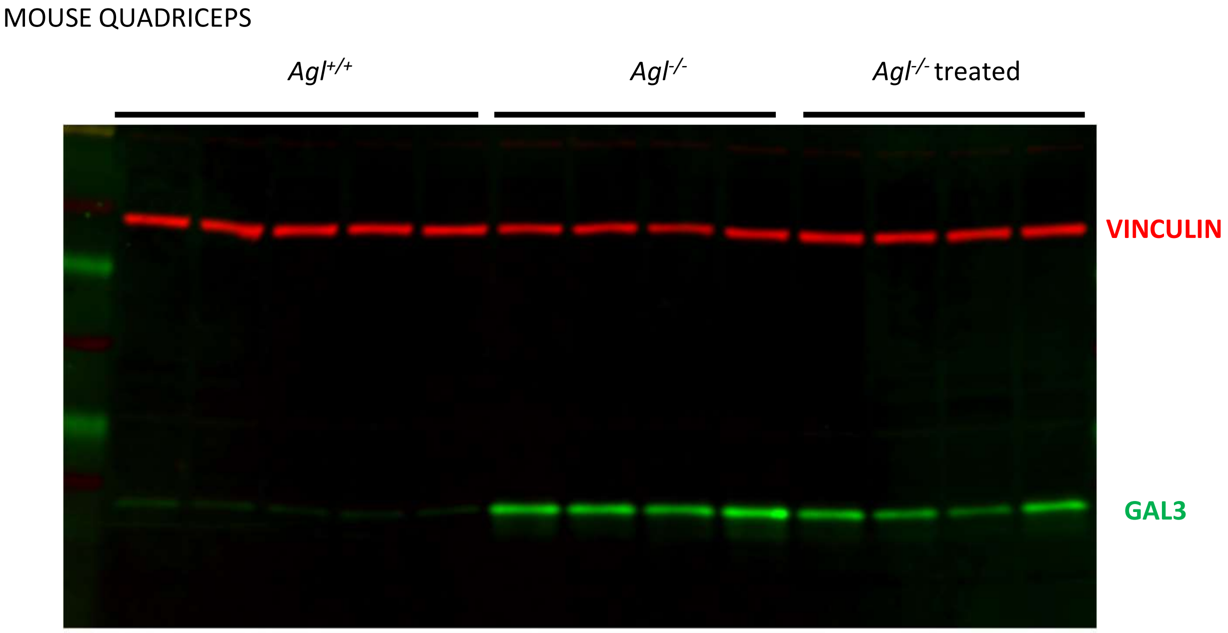

Original Data 2. *LGALS3* up-regulation confirmed at the protein level (GAL3) in mouse quadriceps biopsies. Uncropped Western Blot analysis of GAL3 protein level in *Agl*$^{+/+}$, *Agl*$^{-/-}$, *Agl*$^{-/-}$ treated mouse triceps biopsies. (n=4 or 5 independent mice for each condition).
